# Supplementary material for: CO2 supply modulates lipid remodelling, photosynthetic and respiratory activities in Chlorella species
Source: Plant Cell Environ. 2021 May 17;44(9):2987–3001. doi: 10.1111/pce.14074 (PMC8453743; doi:10.1111/pce.14074)

Figure S1. Dry biomass per cell in *C. vulgaris* and *C. sorokiniana*. Dry weight (DW) per cell in the case of *C. sorokiniana* and *C. vulgaris* in AIR condition (~0.04% CO_2) compared to CO_2 condition (3% CO_2) are reported. Dry biomass per cell was calculated from cell density and total dry weight per liter measured at the end of the growth curve reported in Figure 1. Data are means of 4 replicates and error bars represent standard deviations. Significantly different values in CO_2 versus AIR are indicated by * ($P < 0.05$, n=3).

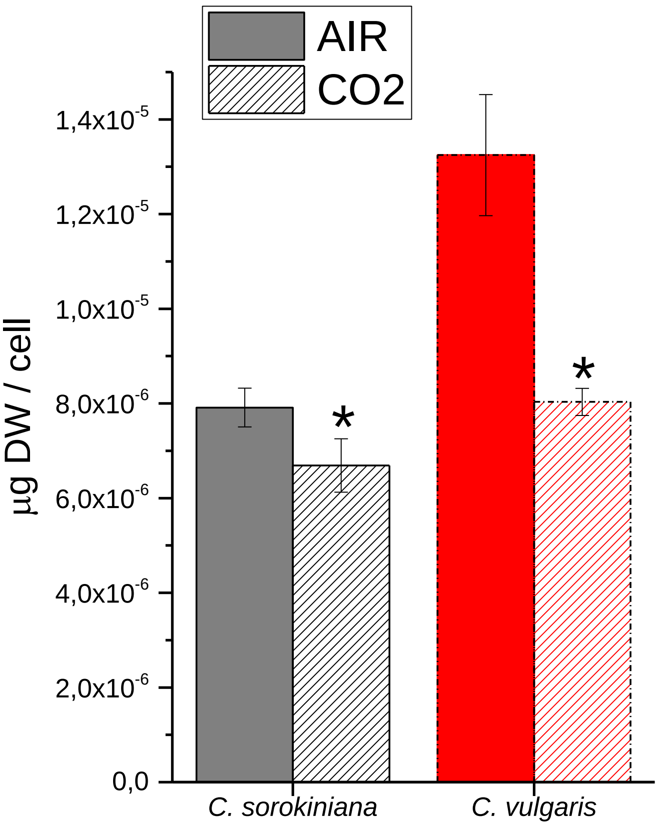

Figure S1. Starch, lipids and protein content per cell in AIR *vs.* CO_2. Relative starch, protein, and lipid content per cell in *C. sorokiniana* (Panel A) and *C. vulgaris* (Panel B) in AIR *vs.* CO_2 condition. Data are means of three biological replicates with standard deviation shown. Significantly different values respectively for starch, protein, and lipids content per cell in CO_2 versus AIR or in *C. vulgaris* versus *C. sorokiniana* are indicated with different letters ($P < 0.05$, n=3).

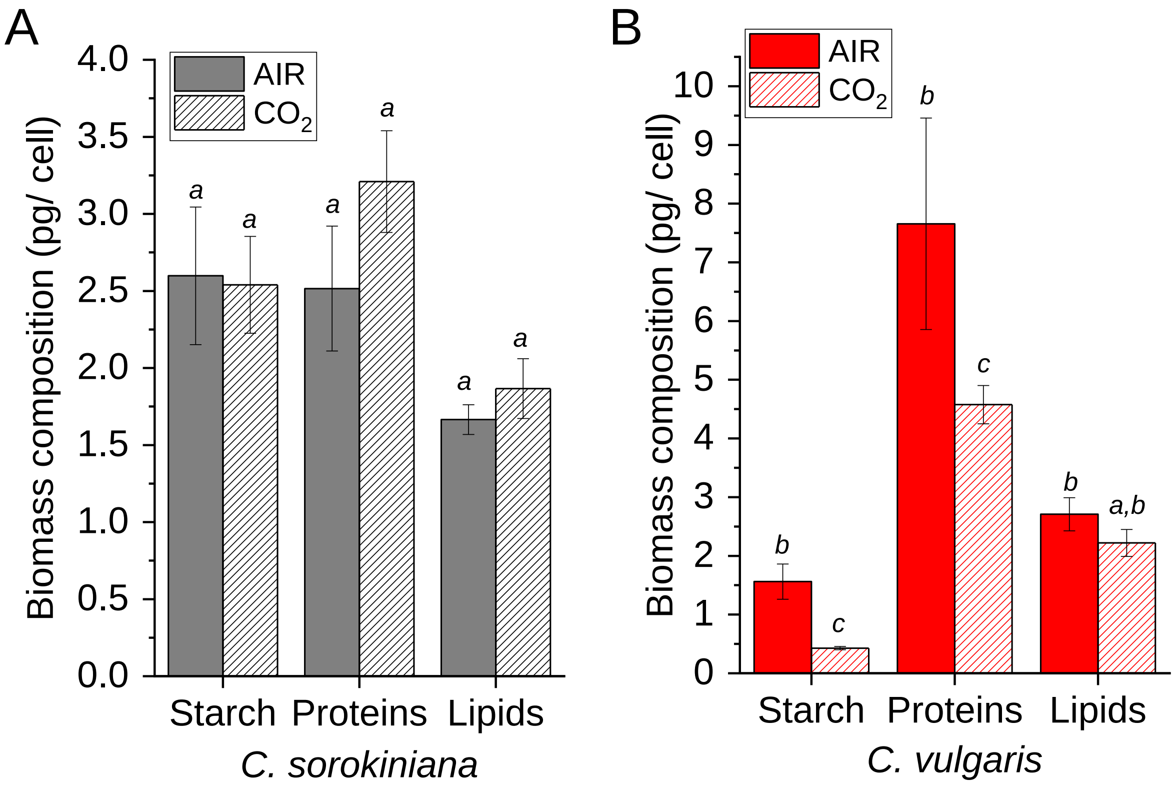

Figure S2. Lipid and fatty acids accumulation per cell in AIR *vs.* CO_2 conditions. Lipids accumulation per cell in *C. sorokiniana* (panel A-C) and *C. vulgaris* (panel D-F) cells grown in AIR or CO_2 conditions. Panel A and D: lipid accumulation per cell in AIR *vs.* CO_2 condition in terms of phospholipids, galactolipids, DGTS and triacylglycerol (TAG). Panel B and E: Fatty acids accumulation per cell obtained by gas chromatography. Panel C and F: Polar lipid accumulation per cell obtained by thin layer chromatography. Data are means of three biological replicates with standard deviation shown. Significantly different values in CO_2 versus AIR are indicated by * ($P < 0.05$). MGDG, monogalactosyldiacylglycerol; DGDG, digalactosyldiacylglycerol; PG, phosphatidylglycerol; PE, phosphatidylethanolamine; PC, phosphatidylcholine; DGTS, diacylglycerol *N*,*N*,*N*-trimethylhomoserine.

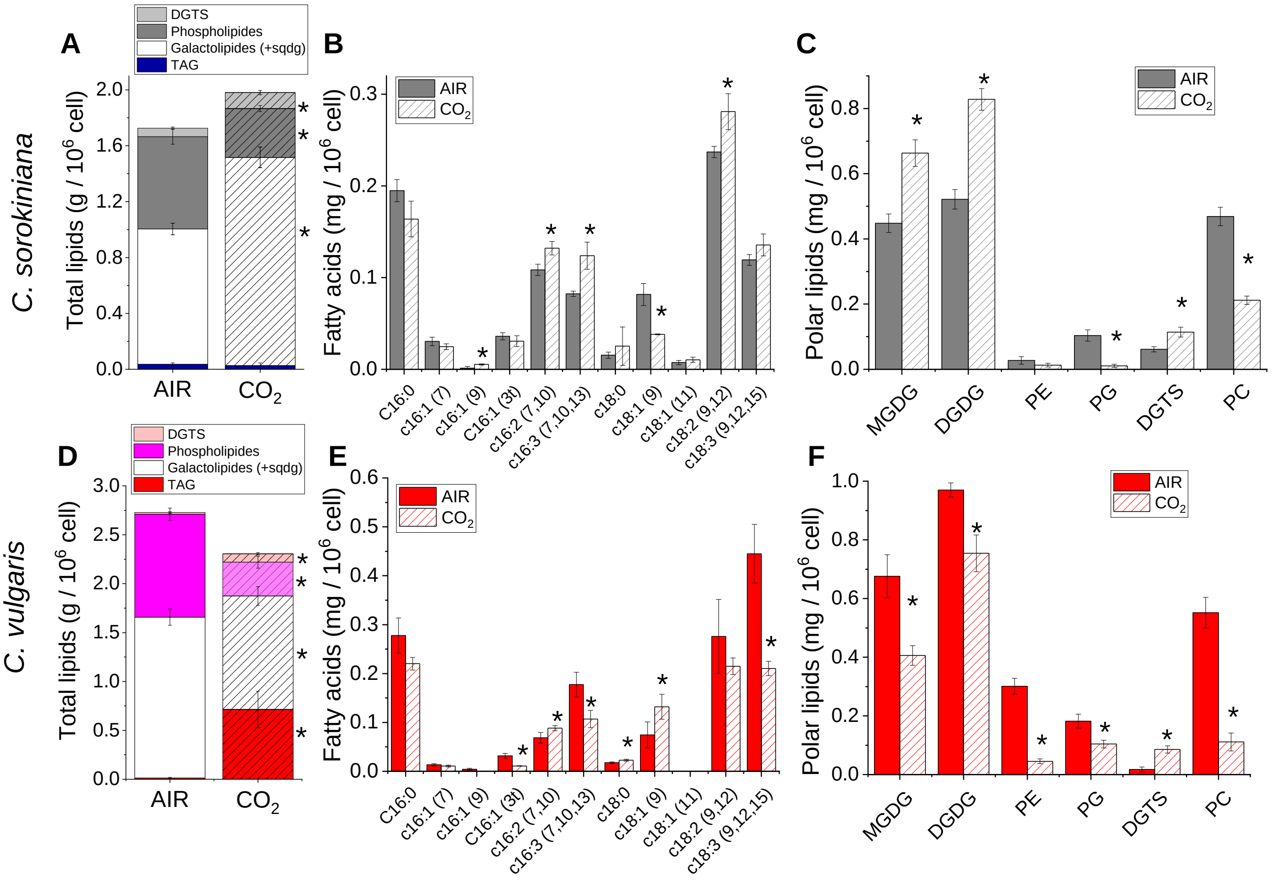

Figure S4. Immunoblot analysis of RUBISCO in *C. vulgaris* and *C. sorokiniana*. Specific antibody recognizing large subunit of RUBISCO (α-RbcL) was used to analyse the relative content of RUBISCO in *C. sorokiniana* (grey colour) and *C. vulgaris* (red colour) in AIR (full colour) or CO_2 (dash colour) condition. Coomassie stained SDS-PAGE gel loaded in parallel with the SDS-PAGE gel used for western blots are reported as loading control. The amounts of chlorophylls loaded for each lane is reported on top of the Figure. Immunoblotting results were then quantified by densitometry and normalized to chlorophyll (chl)per cell ratio to evaluate the RUBISCO content per cell considering chl/cell ratio respectively of $3.7x10^{-7} \pm 0.1x10^{-7}$ and $2.2x10^{-7} \pm 0.2.x10^{-7}$ μg Chl/cell for *C. sorokiniana* cells in AIR and CO_2 conditions, and $3.5x10^{-7} \pm 0. 4x10^{-7}$ and $3.3x10^{-7} \pm 0.2.x10^{-7}$ μg Chl/cell for *C. vulgaris* cells in AIR and CO_2 conditions.

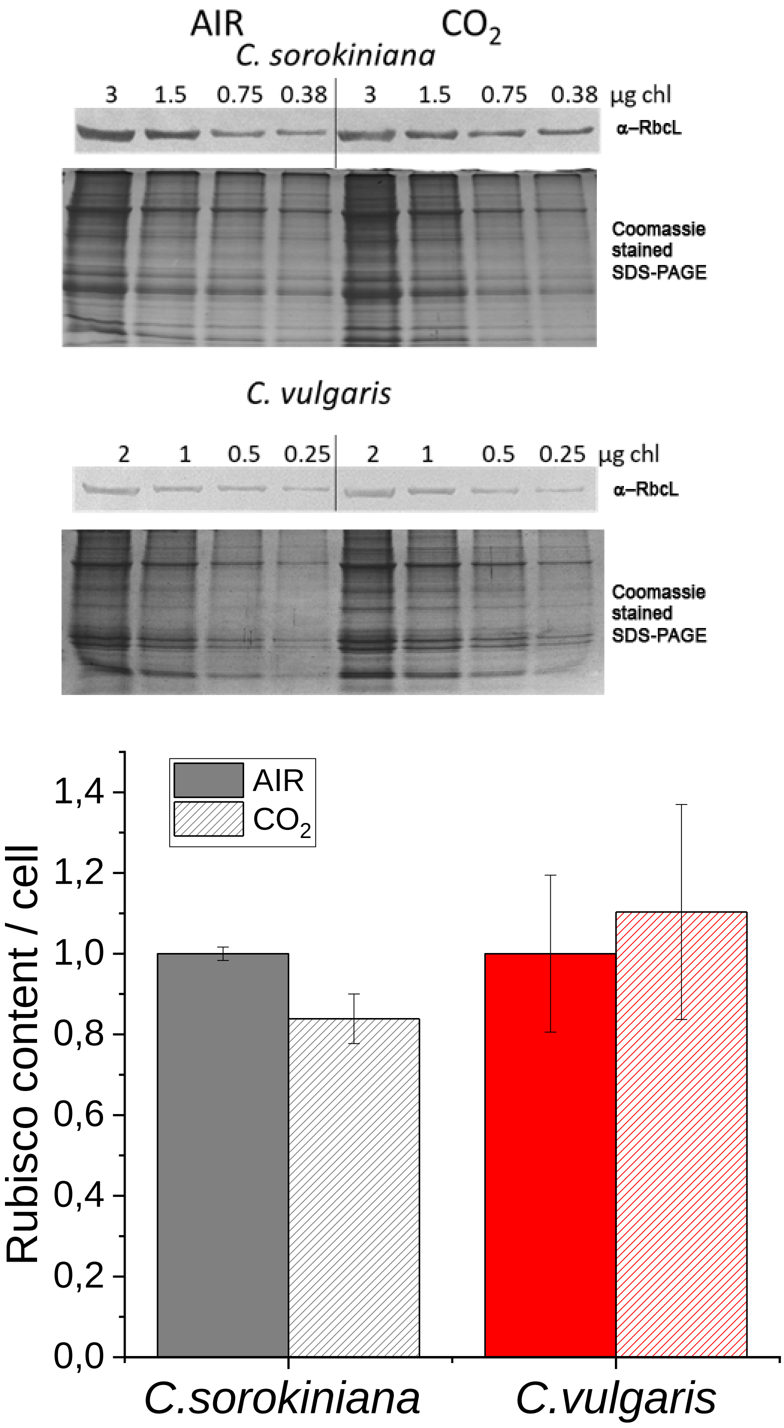

Figure S5. Functional antenna size of PSII in *C. vulgaris* (*Cv*), *C. sorokiniana* (*Cs*) and *C. reinhardtii* (*Cr*) in AIR *vs.* CO_2 conditions. PSII functional antenna size can be estimated from chlorophyll *a* fluorescence emission kinetics in whole cells in limiting light treated with DCMU to inhibit photochemical activity of PSII (A). PSII functional antenna size can be expressed as the reciprocal of the time required to reach 2/3 of the maximum fluorescence emission ($1/\tau_{2/3}$, B). Errors bars are reported as standard deviation. Significantly different values in CO2 versus AIR are indicated by * ($P < 0.05$).

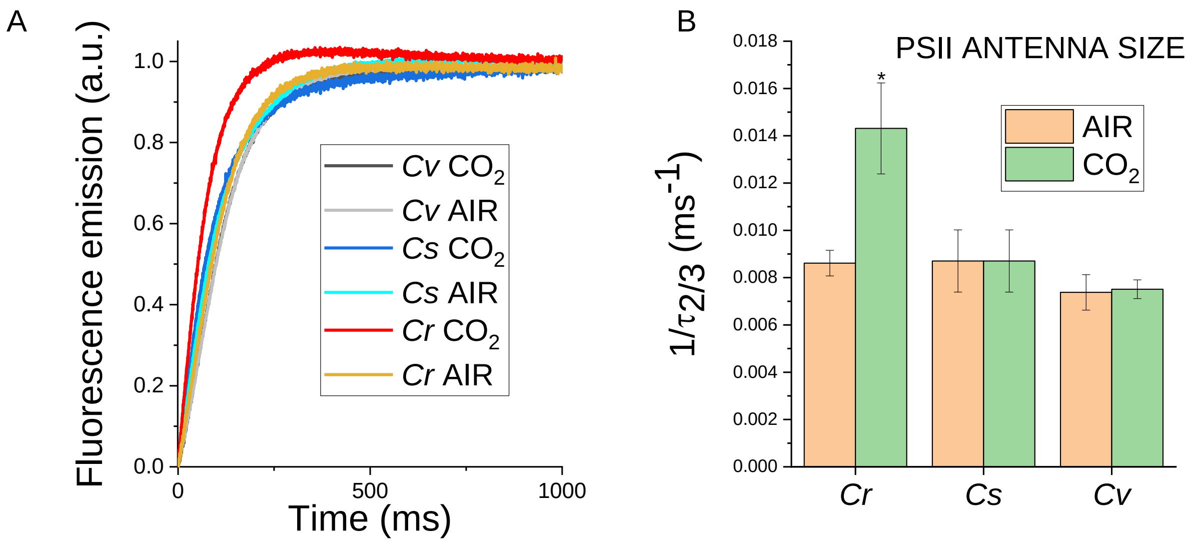

Figure S6. NAB1 BLAST search in *C. vulgaris* and *C. sorokiniana*. e-values and sequence length are reported of the different results obtained. Cold-shock domain (CSD) and RNA-recognition motif (RRM) are indicated respectively in grey and orange as described in the inset.

Species	Protein name	e-value	Sequence length (aa)
C. reinhardtii	Cre06.g268600.t1.2 (**NAB1**)		247
C. vulgaris	g211.t1	3e-30	296
	g111.t1	5e-36	150
	g2171.t1	6e-21	227
	g2239.t1	6e-15	416
	g2238.t1	8e-15	358
C. sorokiniana	CSI2_123000002228-RA	6e-29	120
	CSI2_123000003461-RA	3e-22	219
	CSI2_123000002385-RA	2e-16	882
	CSI2_123000004900-RA	9e-10	291

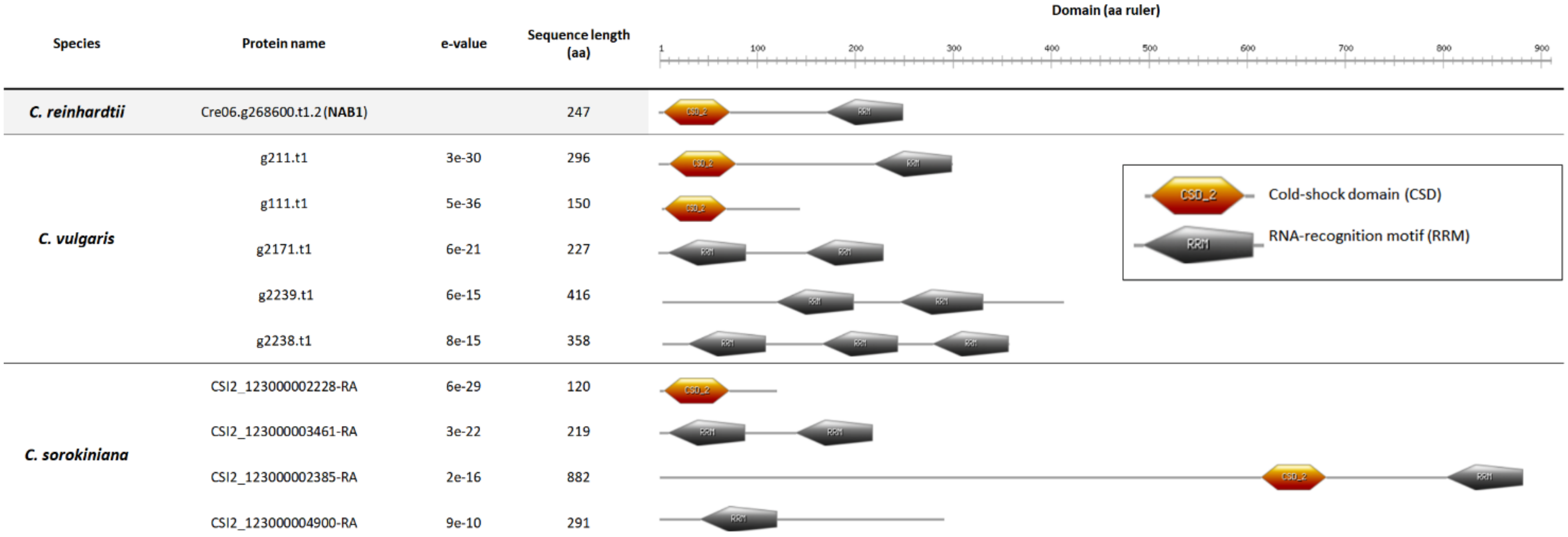

Figure S7. NAB1 immunoblot. Immunoblot analysis of *C. reinhardtii* (A) and *C. vulgaris* (B) cells grown in AIR or in CO_2 condition using an α-NAB1 antibody. Samples were loaded on SDS-PAGE gel at different chlorophyll content as reported in Panel A and B. Coomassie stained SDS-PAGE gel loaded in parallel with the same samples used for western blots are reported as protein loading control. (C-E) Densitometry analysis of immunoblot results. Densitometric results are reported normalized to Chl content (C), or to the cell content (D) considering Chl/cell ratio respectively of $1.0x10^{-6} \pm 1.2x10^{-8}$ and $6.3x10^{-7} \pm 0.2.x10^{-7}$ μg Chl/cell for *C. reinhardtii* cells in AIR and CO_2 conditions, and $3.5x10^{-7} \pm 0.4x10^{-7}$ and $3.3x10^{-7} \pm 0.2.x10^{-7}$ μg Chl/cell for *C. vulgaris* cells in AIR and CO_2 conditions, or normalized to the total protein loaded in the gel determined by densitometry of Coomassie stained SDS-PAGE gel (E). Significantly different values in CO_2 versus AIR are indicated by * ($P < 0.05$, n=3). Data are reported normalized to 1 in the case of the AIR condition for both *C. reinhardtii* and *C. vulgaris.*

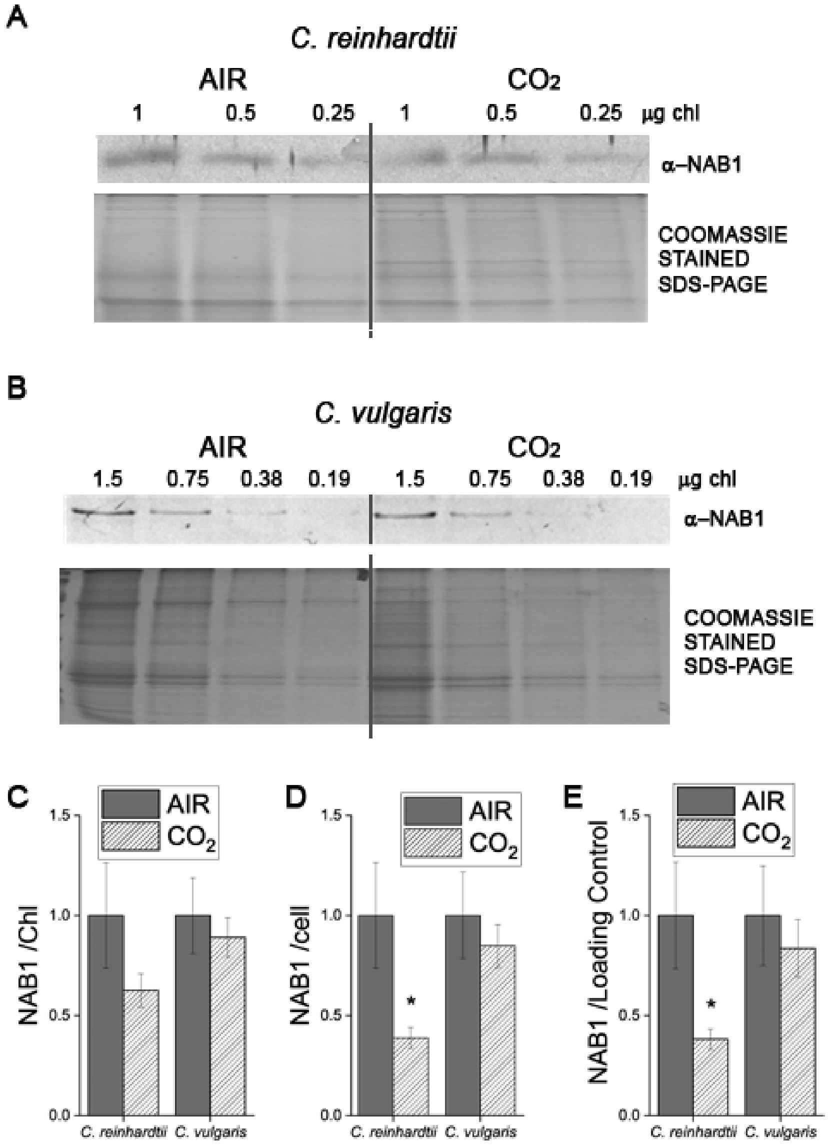

Figure S8 Protein alignment of *C. reinhardtii* NAB1 and *C. vulgaris* g211.t1 predicted protein. Clustal Omega protein alignment of NAB1 from *C. reinhardtii* (Cre06.g268600.t1.2) and the predicted homologous protein in *C. vulgaris* (g211.t1). A 39% percentage of identities was found between the two proteins. Key residues previously identified for the translational repressor activity of NAB1 in *C. reinhardtii* (Cre06.g268600.t1.2) are highlighted in cyan in the case of Arg90 and Arg92, in yellow in the case of Cys181 and Cys226.

```
Cre06.g268600.t1.2      ------MGEQLRQQGTVKWFNATKGFGFITPGGGGEDLFVHQTNINSEGFRSLREGEVVE 54
g211.t1                 MGEPEEPQATERQFGTVKWFNSTKGYGFITSEDCEDEVFVHQSNIETTGYRSLKEGEEVE 60
                                   ** *******:***:****   .  :::****:**:: *:***:*** **

Cre06.g268600.t1.2      FEVEAGPDGRSKAVNVTGPGGAAPEGAPR-------NFRGGGRGRGRARGARGGYAAAYG 107
g211.t1                 FDLVVADDGKKKAFRVTGPDGAPPQGSLQPPPMARAGYVGGPSGRGGGRGYDAAYAAAAA 120
                        *:: .. **:.**..****.** *:*: :       .: **  *** .**  ..**** .

Cre06.g268600.t1.2      YPQM---------------------APVYPGYYFFPADPTGRGRGRGGRGG--------- 137
g211.t1                 AGPFGPYAAGGGRGGGAAGRGAYWGPEAYMGYMGYSMGPPGAPMGPGAYYAAAAAAAAAA 180
                           :                       .* **  :  .* *   * *.  .

Cre06.g268600.t1.2      -AMPAMQGVMPGVAYPG-MPMGGVGME---PTGEPSGLQVVVHNLPWSCQWQQLKDHFKE 192
g211.t1                 TAPPAMFPRGRGGFFPGGRNWAGARPPPPGQPGFSSGLQVVVHNLPWDCTWQQLKDAFIA 240
                         * ***     *  :**    .*.        *  ************.* ****** *

Cre06.g268600.t1.2      W-RVERADVVYDAWGRSRGFGTVRFTTKEDAATACDKLNNSQIDGRTISVRLDRFA     247
g211.t1                 CGDIERADVVFDSRGRSRGFGIVRFPSKDMADTAVNTMNNSTIGGRVVSVRIDRFA     296
                           :******:*: ******* *** :*: * ** :.:*** *.**.:***:****
```

Figure S9. Model of the main metabolic rearrangements of *C. sorokiniana* and *C. vulgaris* cells grown with 3% CO_2. Green/red arrows indicate respectively increase/reduced metabolic flow in condition of high CO_2 availability (3% CO_2) compared to the case of cultivation at atmospheric concentration of CO_2. The width of the arrows is proportional to the possible metabolic flow. Black line indicates similar metabolic flow in AIR vs CO_2 condition. Chloroplast is indicated in green, mitochondria in orange. In this model, both *C. vulgaris* and *C. sorokiniana* grown at high CO2 availability (+CO_2) increase photosynthetic ATP and NADPH production to provide respectively energy and reducing power for carbon assimilation. The main acclimation process at the level of the photosynthetic apparatus includes in *C. sorokiniana*, but not in *C. vulgaris,* reduced LHCII and PSI accumulation compared to PSII. In both species reduced *pmf* was measured, likely due to increased proton release in the stroma by increased ATPase accumulation. In *C. sorokiniana* a similar $NAD(P)^+$/NAD(P)H ratio was measured, which was instead reduced in the case of *C. vulgaris*. The reduced mitochondrial respiration measured in the case of *C. vulgaris* grown at high CO_2 availability suggests a possible increased exchange of reducing power from the mitochondria to the plastids. In *C. vulgaris*, the increased carbon flow because of increased CO_2 availability caused increased proteins and lipids production, with a specific increased of MGDG and DGDG, while in the case of C. vulgaris increased TAG accumulation was observed.

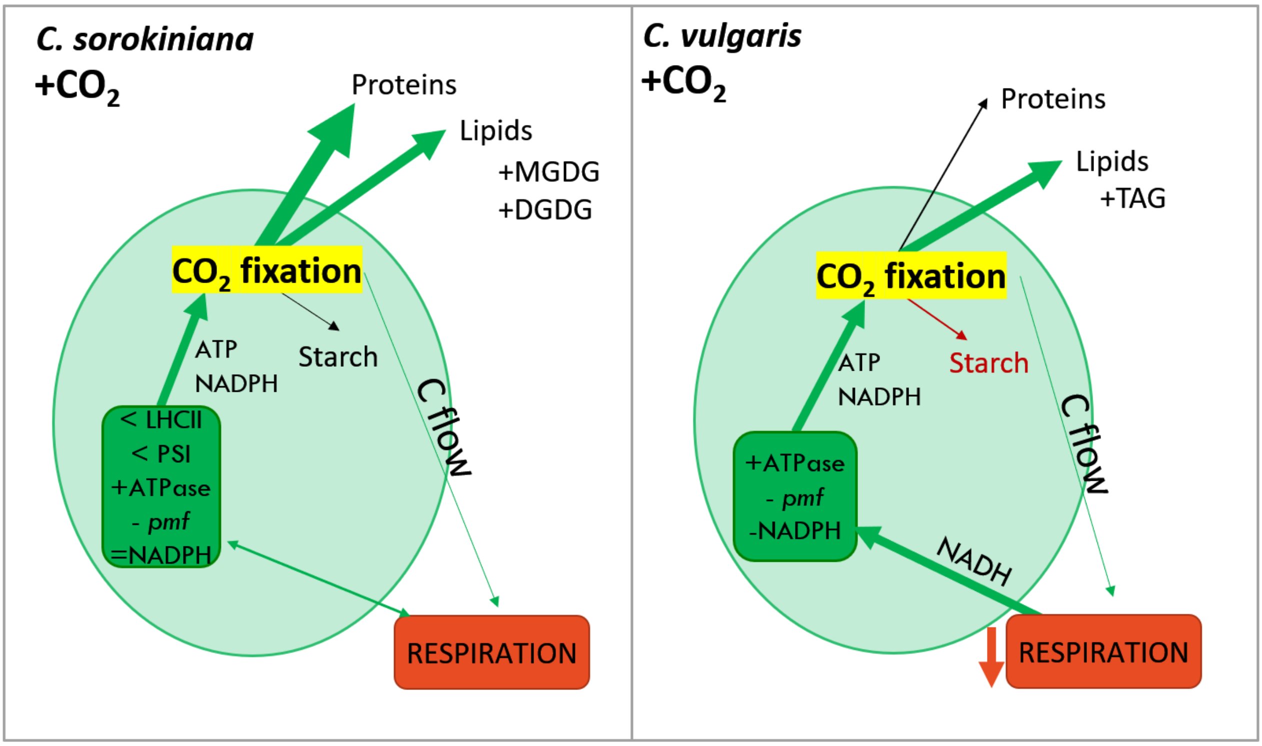

Supplement: Supplementary file 1 — Data S1. Supporting information. [file PCE-44-2987-s001.pdf]
